# Incidence and predictors of respiratory tract infections among birth cohorts in Ethiopia, 2023

**DOI:** 10.1186/s13052-025-01838-7

**Published:** 2025-02-07

**Authors:** Fekade Demeke Bayou, Mastewal Arefaynie, Anissa Mohammed, Abiyu Abadi Tareke, Awoke Keleb, Natnael Kebede, Yawkal Tsega, Bayu Hailemariam Mersha, Abel Endawkie, Shimels Derso Kebede, Kaleab Mesfin Abera, Eyob Tilahun Abeje, Ermias Bekele Enyew, Chala Daba, Lakew Asmare

**Affiliations:** 1 Department of Epidemiology and Biostatistics, School of Public Health, College of Medicine and Health Science, Wollo University, Dessie, Ethiopia; 2 Department of Reproductive and Family Health, School of Public Health, College of Medicine and Health Sciences, Wollo University, Dessie, Ethiopia; 3 Amref Health in Africa, COVID-19 Vaccine/EPI Technical Assistant at West Gondar Zonal Health Department, Gondar, Ethiopia; 4 Department of Environmental Health, College of Medicine and Health Sciences, Wollo University, Dessie, Ethiopia; 5 Department of Health Promotion, School of Public Health, College of Medicine and Health Sciences, Wollo University, Dessie, Ethiopia; 6 Department of Health System and Management, School of Public Health, College of Medicine and Health Sciences, Wollo University, Dessie, Ethiopia; 7 Farah Wais Dule General Hospital, Somali, Ethiopia; 8 Department of Health Informatics, School of Public Health, College of Medicine and Health Sciences, Wollo University, Dessie, Ethiopia; 9 Department of Epidemiology and Biostatistics, Institute of Public Health, College of Medicine and Health Science, University of Gondar, Gondar, Ethiopia

**Keywords:** Incidence, Respiratory tract infections, Predictors, Ethiopia

## Abstract

**Background:**

Respiratory tract infection (RTI) has been a predominant health problem worldwide, including Ethiopia. It is one of the major causes of under-five mortality in Ethiopia, accountable for 18% of total deaths. Nationwide studies on the incidence of respiratory infections and maternal risk factors are limited. Hence, this study was aimed to determine the incidence and predictors of respiratory infections among infants aged less than six months in Ethiopia, 2023.

**Methods:**

This was a retrospective follow up study using data from Performance Monitoring for Action Ethiopia (PMA Ethiopia). A two-stage cluster sampling technique was used to select a total 2,246 eligible study participants from 206 enumeration areas. A Cox proportional hazards regression model was used to identify independent predictors of respiratory tract infection incidence. Hazard ratios with 95% confidence intervals and p value < 0.05 were used to declare statically significant associations between variables.

**Results:**

The incidence rate of respiratory tract infections among birth cohorts was 22.99 per 1000 infant weeks of observation. Infants born from mothers who sustained convulsion during labor had nearly doubled [AHR = 1.65, 95%CI (1.20, 2.28)] hazard or risk of developing respiratory tract infections than their counterparts. Similarly, infants born from mothers having prolonged labor (more than 12 h) had one and half times [AHR = 1.48, 95%CI (1.13, 1.93)] increased risk of developing respiratory tract infections as compared to their counterparts.

**Conclusion:**

The incidence of respiratory tract infections is a significant public health concern in Ethiopia. Infants born to mothers with prolonged labor and convulsions need closer monitoring for early signs of respiratory tract infections.

**Supplementary Information:**

The online version contains supplementary material available at 10.1186/s13052-025-01838-7.

## Introduction

Respiratory tract infections are infections in any area of the respiratory tract, including the nasal cavity, sinuses, pharynx, larynx, epiglottis, trachea, bronchi, and lungs, with symptoms lasting for 30 days or less. Respiratory tract infections range from mild infection to fatal disease, depending on the causative pathogen, as well as environmental and host factors. The mode of transmission of most RTIs is through droplets, infectious respiratory aerosols, or contact with others with an infection [1]. The common manifestations include cough, runny nose, sore throat, high fever, chills and other serious symptoms are difficulty breathing, dizziness, low blood oxygen level and loss of consciousness [2].

Respiratory tract infections (RTIs) can be classified in to two: upper and lower RTIs. The upper respiratory tract infections include croup, whooping cough, ear infection, throat infection, rhinitis and cold. Lower respiratory tract infections include bronchitis, bronchiolitis, pneumonia and chest infections. Respiratory tract infections are common in early life, which have short-term consequences, but also affect the development of both the respiratory and immune systems [3]. In 2021, the global incidence rate of upper respiratory tract infections (URTIs) was 162,484.8 per 100,000 people. The highest rates were observed in children under 2 years old, while the most episodes occurred in children aged 5 to 9 years [4].

Ethiopia has reduced child mortality, however, the decline in neonatal mortality was not proportional and currently 42% of childhood deaths in the country occur within the first 28 days of life. Respiratory tract infections are major causes of under-five mortality in Ethiopia; being accountable for 18% of the total deaths [5]. In 2019, an estimated 6, 628, 674 (6, 108, 786 − 7, 230, 986) new cases of RTIs occurred in Ethiopia. Out of the total RTIs episodes, 22% (1, 448, 680) of new cases occurred among children younger than 5 years, yielding an annual incidence rate of 8,685 per 100,000 children (6, 895 − 10,790) [6].

Ethiopia had been implementing child survival programs in the last decades by adopting integrated management of newborn and childhood illnesses (IMNCI) guidelines. Significant improvements in coverage had been observed primarily in preventive interventions including immunizations, vitamin A supplementation, insecticide-treated nets, and water and sanitation [5]. Scientific evidences regarding the level of new morbidity on such prevailed health problems are paramount to decide on future areas of intervention. However, in Ethiopia, nationwide studies on the incidence of RTIs and maternal risk factors are limited. Moreover, existing studies focused on socio-demographic and environmental factors, and did not addressed maternal risk factors of RTIs including pregnancy, delivery and postnatal related complication, mode of delivery, and antenatal care services. Hence, we were aimed to address this information gaps.

## Methods

### Study setting

The study was conducted in six regions of Ethiopia including: Addis Ababa, Tigray, Afar, Amhara, Oromia and South Nation, Nationalities and People (SNNP) regions.

### Study design

Retrospective follow up study was conducted.

### Population

All infants and their mothers residing in households, located within the six regions were the source population. Infants aged less than six months and their mothers who met the eligibility criteria were the study population.

### Eligibility criteria

All women aged between 15 and 49 years who gave birth during the panel and their newborns in the selected regions were eligible for this study. Women and newborns with incomplete data on the outcome variable and predictors were excluded from the final analysis in this study.

### Sample size and sampling procedure

A total of 206 enumeration areas (EAs), located within the six regions described above were selected from the master sample frame of the Central Statistical Agency (CSA) of Ethiopia. All newborns and their mothers residing in households, located within the Panel survey regions were participated in this study. From 2,868 women involved in the Panel survey, a total of 2246 women with their newborns were eligible for data analysis.

### Data sources

This study used the data from Performance Monitoring for Action Ethiopia (PMA Ethiopia). PMA Ethiopia is a five-year project implemented in collaboration with Addis Ababa University, Johns Hopkins University, and the Federal Ministry of Health. It measures key reproductive, maternal and newborn health (RMNH) indicators. In this project, longitudinal data are collected between 2021 and 2023 in six, predominantly agrarian regions including: Addis Ababa, Tigray, Afar, Amhara, Oromia and South Nation, Nationalities and People (SNNP) regions.

### Data management and analysis

The data were exported from Microsoft excel to SPSS, cleaned, coded and analyzed using SPSS version 25. Descriptive statistics including frequency, percent, mean and standard deviation were used to present the characteristics of study participants. A Cox proportional hazards regression model was used to identify independent predictors of respiratory infection incidence. The global test was checked for cox proportional hazards assumption, the result revealed that the assumption was met with a p-value of 0.18. Hazard ratios and their 95% confidence intervals was calculated for each predictor variable. A p value of < 0.05 was used to declare statistical significance.

## Results

### Characteristics of the study participants

In this study a total of 2246 newborns were included, with almost the same proportion between male and female, ratio m: f of 1:1. Their age ranges from 2 to 26 weeks with median age of 8 weeks (interquartile range = 15 − 7 weeks). Regarding residency, nearly a quarter (24.4%) of the participants were living in Oromia region, 23.2% in South Nation Nationalities and Peoples (NNPR) region, 17.6% in Amhara region, 17.2% in Tigray region, 9.2% in Addis Ababa and 8.3% in Afar region. More than one-thirds (38.2%) of births took place by non-skilled attendant while the remaining took place by skilled attendants (58.1% at health centers or public hospitals, 2.4% at private clinics or hospitals, 0.8% at non-governmental (NGO) facilities and 0.4% at health posts).

### Incidence of respiratory tract infection

From 2, 246 birth cohorts followed for a total of 23, 840 infant weeks, 548 infants had developed the outcome of interest (respiratory tract infections). The median time of developing RTI was 23.0 weeks. Hence, the incidence rate of respiratory (including upper and lower) tract infection symptoms among birth cohorts was 22.99 per 1000 infant weeks of observation. This implies, on average, 23 out of 1000 newborns would develop respiratory tract infection per a week. Regarding respiratory symptoms, 463 (20.6%) had cough, 129 (5.7%) had fever, 60 (2.7%) had sore throat or tonsillitis, 44 (2.0%) had fast breathing, 39 (1.7%) had difficulty breathing, and 10 (0.4%) had chest in drawing.

### Predictors of the incidence of respiratory tract infection

Predictor variables were selected based on their clinical relevance and preliminary assessment for association using bi-variable regression model. Accordingly, region, born from mother having abnormal vaginal discharge during pregnancy, maternal intake of iron during pregnancy, being seen by health extension workers, delivery (labor) complication including convulsion, prolonged labor (more than 12 h), mal-presentation, and premature rupture of membrane were assessed for their association with the outcome variable using cox proportional hazards regression model. However, only three variables: region, having convulsion during labor and prolonged labor were significantely associated with the incidence of respiratory tract infection during the first few weeks of the infancy period. Infants from Amhara and SNNP regions had 1.48 (95%CI: 1.02, 2.12) and 1.69 (95%CI: 1.20, 2.38) times higher risk of developing RTIs than those from Tigray region respectively. Infants born from mother sustained convulsion during labor had nearly doubled [AHR = 1.65, 95%CI (1.20, 2.28)] hazard or risk of developing respiratory tract infections than their counterparts. Similarly, infants born from mothers having prolonged labor (more than 12 h) had one and half times [AHR = 1.48, 95%CI (1.13, 1.93)] increased risk of developing respiratory tract infections as compared to their counterparts (Table 1).

**Table 1:** Predictors of the incidence of respiratory infection among birth cohorts in Ethiopia, 2023

Variables		Respiratory infection		CHR (95%CI)	AHR (95%CI)
		Yes: n (%)	No: n (%)		
Regions	Tigray	72(18.6)	315(81.4)	1	1
	Addis Ababa	34(16.4)	173(83.6)	0.78 (0.52, 1.169)	0.86(0.55, 1.34)
	Afar	35(18.8)	151(81.2)	0.99(0.66, 1.48)	1.05(0.45, 2.49)
	Amhara	105(26.5)	291(73.5)	1.53(1.13, 2.06)	1.47(1.02, 2.12)
	Oromia	126(23.0)	422(77.0)	1.27(0.95, 1.70)	1.09 (0.76, 1.58)
	SNNPR	176(33.7)	346(66.3)	1.96 (1.49, 2.57)	1.69 (1.20, 2.38)
Mode of delivery caesarean section	Yes	29(17.7)	135(82.3)	0.76 (0.52, 1.12)	0.88 (0.56, 1.33)
	No	276(22.8)	937(77.2)	1	1
Convulsion during labor	Yes	92(39.1)	143(60.9)	1.97 (1.58, 2.47)	1.65(1.20, 2.28)
	No	456(22.7)	1555(77.3)	1	1
Prolonged labor (> 12 h)	Yes	111(30.7)	251(69.3)	1.47 (1.19, 1.81)	1.48(1.13, 1.93)
	No	437(23.2)	1447(76.8)	1	1
Mal- presentation	Yes	23(25.0)	69(75.0)	0.98 (0.64, 1.49)	0.88 (0.53, 1.48)
	No	525(24.4)	1629(75.6)	1	1
Premature rupture of membrane	Yes	16(30.8)	36(69.2)	1.31(0.80, 2.15)	0.91 (0.48, 1.75)
	No	532(24.2)	1662(75.2)	1	1
Abnormal vaginal discharge during pregnancy	Yes	17(36.2)	30(63.8)	1.97 (1.21, 3.188)	1.79(0.89, 3.61)
	No	531(24.1)	1668(75.9)	1	1
Taken iron during pregnancy	Yes	359(23.5)	1169(76.5)	0.83 (0.70, 0.99)	0.89 (0.67, 1.18)
	No	189(26.3)	529(73.7)	1	1
Seen by health extension workers	Yes	163(25.0)	489(75.0)	1.11(0.92, 1.33)	0.86(0.65,1.13)
	No	385(24.2)	1209(75.8)	1	1

CHR: crude hazard ratio, AHR: adjusted hazard ratio, HC: health center, SNNP: south nation nationalities and people’s region

## Discussion

This study was aimed to assess the incidence of respiratory tract infections and risk factors during the first six months of life among birth cohorts in Ethiopia. The study found that the incidence of the outcome of interest was 22.99 per 1000 infant weeks of observation. Maternal complications like prolonged labor and convulsion during delivery had increased the risk of developing respiratory tract infections among newborns and infants during their earlier lives. The incidence of respiratory tract infection had also a significant difference across regions of Ethiopia.

In this study nearly 23 infants experiencing respiratory symptoms for every 1,000 infant-weeks of observation, underlining the frequency of these infections in this age group. The incidence rate of respiratory tract infection in early life of infants found by this study was higher than the figure reported by Zhou et al., who found an incidence rate of 2.8 per 1000-person months in Quebec City, Canada [7]. Similarly, our finding was lower than the report from Brazil, which found 0.51 episodes per 100 children-months and 3.10 episodes per 100 children-months of lower respiratory airway infection [8]. The possible explanation for the observed discrepancy might be difference in sociodemographic characteristics of the study populations, health systems, and the outcome measurement. For example, the above study investigated the incidence rate of lower respiratory tract infections after following children from birth up to the age of 24 months.

On the other hand, the incidence rate of RTI reported in this study was lower than the finding of similar situations in Bangladesh, which found an incidence rate of 330 infections per 100 infant-years [9]. The possible justification for the observed difference might be seasonal variation, study population differences and health system related variations like immunization coverage, basic health services and health literacy. The above study found the peak incidence of acute respiratory tract infections (ARTI) during rainy and cold season among 2–6 months aged children.

The figure in our study revealed that respiratory tract infections are still the leading cause of morbidity and mortality among infants in Ethiopia. Recurrent respiratory infections in early life can have negative consequences for infant development, potentially impacting lung function, growth, and cognitive development [3, 10]. Understanding the incidence and prevalence of these infections is crucial for designing interventions to mitigate the risks. This high incidence rate underscores the importance of public health efforts aimed at preventing and managing these infections.

In this study Infants born from mother sustained convulsion during labor had nearly doubled the hazard or risk of developing respiratory tract infections than their counterparts. One reason for this condition might be because of maternal convulsions during labor can cause significant physiological stress on both the mother and the fetus. This stress response can lead to hormonal imbalances, impaired placental blood flow, and fetal hypoxia, all of which can negatively impact the infant’s immune system and increase susceptibility to infections [11]. The second possible way might be; during convulsions, the mother may lose consciousness and control over her airway, increasing the risk of aspiration of meconium or amniotic fluid by the infant. This aspirated material can irritate the airways and predispose the infant to pneumonia [12]. Moreover, convulsions during labor can be a sign of underlying maternal or fetal complications, such as preeclampsia, eclampsia, or placental abruption. These conditions can also lead to neonatal encephalopathy, which can impair the infant’s ability to clear secretions and fight infections. Beside this, as convulsing mothers are more likely to take anticonvulsants, those medications administered to the mother during or after convulsions can cross the placenta and enter the infant’s circulation. Some of these medications can suppress the infant’s immune system and increase the risk of infections [13].

The present study found a concerning association between prolonged labor (greater than 12 h) and an elevated risk of respiratory tract infections (RTIs) in infants. Infants born after such labors were one and a half times more likely to develop RTIs compared to those born from shorter labors. Several mechanisms might explain the observed association: (I) Prolonged labor can expose the fetus to hypoxia, acidosis, and inflammation, potentially compromising their immune system and making them more susceptible to respiratory pathogens [14]. (II) Prolonged exposure to amniotic fluid, laden with inflammatory mediators and potential pathogens, might increase the risk of aspiration and subsequent RTIs [15]. (III) Mothers experiencing prolonged labor might have a higher risk of underlying infections, which could be transmitted to the infant during birth, increasing their susceptibility to RTIs [16].

This study might have the following limitations: since the current study uses secondary data sources, it missed important variables to see their association with the outcome variable. The other limitation was, as the study included selected regions of Ethiopia, it might not represent the national level incidence of respiratory infections.

### Conclusion and recommendation

The incidence of respiratory infection is a significant public health concern in Ethiopia. Our findings suggest that infants born to mothers with labor convulsions may benefit from closer monitoring for early signs of RTIs. Clinicians should be aware of this association and consider additional preventive measures, such as early initiation of breastfeeding to support immune development and prompt administration of appropriate antibiotics if an RTI is suspected. Further research is needed to elucidate the specific mechanisms underlying this association and to identify potential interventions to reduce the risk of RTIs in infants born to mothers with labor convulsions. Prospective studies with larger sample sizes are needed to confirm our findings and to explore the influence of specific maternal and fetal factors on the risk of RTIs. Additionally, research is needed to investigate the long-term consequences of RTIs in this vulnerable population.

## Electronic supplementary material

Below is the link to the electronic supplementary material.

Supplementary Material 1

## Data Availability

The dataset used in this study is available at the principal investigator.

## Abbreviations

AHR: Adjusted Hazard Ratio
ARI: Acute Respiratory infection
CHR: Crude Hazard Ratio
CI: Confidence Interval
RTI: Respiratory Tract Infection
