# Supplementary material for: Incidence and predictors of respiratory tract infections among birth cohorts in Ethiopia, 2023
Source: Ital J Pediatr. 2025 Feb 7;51:32. doi: 10.1186/s13052-025-01838-7 (PMC11806740; doi:10.1186/s13052-025-01838-7)
Supplement: Supplementary file 1 — Supplementary Material 1 [file 13052_2025_1838_MOESM1_ESM.docx]

**Cover letter**

**To:** **Italian Journal of Pediatrics**

**From:** Fekade Demeke Bayou (MPH in Epidemiology, Lecturer at School of Public Health, College of Medicine and Health Sciences, Wollo University, Dessie, Ethiopia)

**Issue : Sending cover letter for new manuscript submission**

I am Fekade Demeke Bayou, the principal investigator of the manuscript entitled “**Incidence and Predictors of Respiratory Infections among Birth Cohorts in Ethiopia: Retrospective Cohort Study**” we believed that the finding of this study will help to update scholars on the incidence and predictors of RTI in Ethiopia. It will also serve as an input for decision makers and planners working to address child health. It can also be used as a base line data for further investigations by researchers dealing on related discipline. There is no previously published work related to the current study. The type of article; this is an original research. There is no prior interaction with this journal regarding the submitted manuscript. Any academic editor can handle this manuscript as per journals policy of reviewing process and we have no opposition to any reviewer.
